# The N^th^ Wave of COVID-19: Will It Be Alzheimer’s?

**DOI:** 10.1017/cjn.2021.224

**Published:** 2021-09-23

**Authors:** Donald F. Weaver

**Affiliations:** Krembil Brain Institute, University Health Network, Toronto, ON, Canada; Departments of Medicine and Chemistry, University of Toronto, Toronto, ON, Canada

**Keywords:** Alzheimer’s disease, Dementia, Cytokine, Innate immune, COVID-19

Coronavirus disease 2019 (COVID-19) is a multi-organ disease caused by severe acute respiratory syndrome coronavirus 2 (SARS-CoV-2), a zoonotic coronavirus first described in late 2019; on March 11, 2020, the World Health Organization declared the COVID-19 outbreak a Global Pandemic. This pandemic has subsequently undergone first, second, third, and fourth waves with a possibility of ongoing episodic exacerbations (to “The N^th^ Wave”) as COVID-19 transitions from pandemic to endemic. Based on an analysis of evolving evidence, it is reasonable to postulate that dementia or Alzheimer’s disease (AD) could emerge as a long-term consequence of COVID-19. An N^th^ wave of COVID-19 presenting as AD or other types of dementia has major public health implications, sufficient to warrant detailed monitoring and study.

Although the nature of future clinically relevant COVID waves is dependent upon a range of variables, the recent recognition of Long-COVID raises the possibility that forthcoming waves may be an emergence of new symptoms from previous infections, rather than from new infections caused by evolving viral variants. Such delayed symptom occurrence has precedence in infectious diseases arising from microorganisms such as *Treponema pallidum* (general paresis in tertiary neurosyphilis), *Borrelia burgdorferi* (encephalomyelitis in late Lyme Disease), and varicella zoster (herpes zoster shingles as a belated re-emergence of the Chicken Pox virus). Alternatively, delayed symptoms may also arise from the downstream immunotoxic effects of the original infection rather than from viral persistence – a possibility which has precedence from diseases such as the 1918 H1N1 influenza A pandemic in which encephalitis lethargica may have later led to post-encephalitic Parkinsonism (though the precise pathogenesis remains debated).^1^ These time-honored examples of delayed symptoms from infections, combined with newly emerging evidence regarding SARS-CoV-2’s chronic impact on brain, raise the possibility of COVID-19 as a risk for later life cognitive decline, dementia, or even AD. The consideration that COVID-19 might culminate (after a latent phase of undetermined duration) in an N^th^ Wave manifesting as AD is suggested by diverse accumulating data, encompassing the biological properties of SARS-CoV-2 and the clinical features of COVID-19.

## SARS-CoV-2 Is a Neurotropic Virus

Many coronaviruses are neurotropic (having affinity for neural tissue) with β-coronaviruses such as SARS-CoV-2 being well-known to invade the human CNS. SARS-CoV-2 RNA has been detected in cerebrospinal fluid (CSF) and in *post mortem* brain tissue.^2^ This neuroinvasion is effected via several routes including olfactory nerves, splanchnic nerves, and brain vascular endothelium.^3^ In addition, in SARS-CoV-2 the virus’s spike glycoprotein avidly binds angiotensin-converting enzyme 2 (ACE2) with subsequent protease-catalyzed blood-brain barrier damage further facilitating brain invasion.^4^ However, it is also necessary to ascertain if this neurotropism translates into clinically significant neurological symptoms.

## COVID-19 Is a Neurological Disease

SARS-CoV-2 neurotropism is now recognized as clinically relevant.^5^ Although COVID-19 was initially regarded as principally a respiratory disease, neurological symptoms including headache, anosmia, and hypogeusia often preceded pulmonary symptoms; furthermore, the majority of severely afflicted COVID-19 patients exhibit alterations in consciousness, cognition, or behavior, during the course of the disease and often independent of their ongoing cardiorespiratory involvement.^6^ More recently, delirium and cognitive dysfunction have been identified as sole symptoms of SARS-CoV-2 infection with the incidence of delirium exceeding 80% in moderately-severely ill patients.^7^ As recognition of COVID-19 as a neurological disease has evolved, a wider range of sensorimotor and cognitive symptoms have been documented. For example, new-onset seizures are common with epileptiform discharges being detected in nearly half of COVID-19 patients in intensive care.^8^ Magnetic resonance imaging supports these clinical observations, revealing cortico-subcortical edema and/or atrophy, parenchymal micro- and macro-hemorrhages, white matter demyelinating changes, and leptomeningeal enhancement; neuropathological examination is likewise supportive, revealing pan-encephalitis and diffuse petechial hemorrhage of the entire brain in some patients, as well as perivascular and interstitial encephalitis in others.^9^

## COVID-19 Is an Immunological Disease

The mechanistic link connecting the biological presence of SARS-CoV-2 in brain with clinically relevant brain dysfunction is provided in part by the immune system; thus, COVID-19 is not only a neurological disease, it is also an immunological disease.^10^ In some individuals, SARS-CoV-2 infection triggers a massive release of pro-inflammatory cytokines (*e.g.,* Interleukin (IL)-6, IL-8, tumor necrosis factor (TNF)-α), chemokines, and other inflammation signals in brain leading to activation of microglia, which in turn promotes neuroinflammation and neuronal death.^11^ Of note, the immunopathy of COVID-19 disproportionately (and atypically within the context of chronic illness) involves the innate component of the immune response, with innate immunity being that historically neglected, evolutionarily primitive component of immunology. Specifically, the neuropathology of SARS-CoV-2 infection is enabled by potent immunoevasory mechanisms, which target multiple aspects of innate immunity, leading to induction of hypercytokinemia (*i.e.,* a “cytokine storm” from dysregulated pro-inflammatory cytokine release), impairment of interferon responses, and suppression of antigen presentation.^11^ In addition, pro-inflammatory cytokines increase oxidative stress, which damages cellular membranes and downregulates surface expression of excitatory amino acid transporters, causing elevated glutamate leading to excitotoxic neuronal necrosis and the induction of apoptotic pathways. Because COVID-19 may involve a massive release of inflammatory signals in brain, there are both short-term effects on attention and concentration (delirium) and the potential for long-term effects on memory and cognition (dementia). This involvement of the immune system opens the door to chronic, persistent pathologies extending beyond the timeline of the original acute illness.

## Long-COVID

Long-COVID (Post-COVID-19 syndrome) is a recently proposed collective term denoting persistence of symptoms in those who have recovered from acute SARS-CoV-2 infection.^12^ People experiencing Long-COVID (“long haulers”) experience symptoms include "brain fog", delirium, cognitive dysfunction, depression, fatigue, insomnia, anxiety, dyspnea, cough, palpitations, intermittent fevers, and gastrointestinal symptoms; brain fog is not a recognized diagnostic entity and refers to a person’s self-perceived cognitive deficit occurring because their thought processes have become “fuzzy and sluggish”.^13^ Long-COVID symptoms can persist for months (ongoing studies may ultimately suggest years) and can range from mild to incapacitating, with new symptoms arising well after the time of initial infection. One survey showed that approximately 35% of people who had tested positive for SARS-CoV-2 experienced a range of symptoms that lasted longer than three weeks.^13^ Although its mechanism remains multifactorial and incompletely elucidated, damage from the immune response and its associated inflammation are important contributors. Since COVID-19 has been a recognized disease entity for less than two years, the long-term consequences and ramifications of Long-COVID syndrome remain to be more fully discerned.

## Alzheimer’s Is an Immunological Disease of Brain

The concern that COVID-19’s N^th^ Wave might manifest as dementia, Alzheimer’s disease is supported by a growing body of evidence implicating the role of immunity, particularly innate immunity, in the pathogenesis of AD. Clinically, AD manifests as progressive decline in information processing domains, including memory, cognition, concentration, and executive function; pathologically, AD is characterized by parallel neurotoxic immuno-inflammation and cytotoxic protein oligomerization of Aβ/tau, culminating in interconnected, concomitant immunopathic (pro-inflammatory), and proteopathic (protein misfolding) pathogeneses.

Innate immune activation is an early event in AD pathogenesis, possibly occurring 20-30 years prior to presentation of the first symptoms. This activation may be triggered by pathogen-associated molecular patterns (PAMPs) which induce preliminary pro-inflammatory cascades, eliciting formation of the innate inflammasome with subsequent cytotoxic pro-inflammatory cytokine release.^14^ PAMPs are small molecular motifs within infectious microbes that are recognized by host pattern recognition receptors instigating activation of the innate immune system. A vast array of different types of molecules across a diversity of viral and bacterial microbes can serve as PAMPs; long-past infections from several microbes have already been proposed as triggers of AD and include human herpesviruses and most recently *Porphyromonas gingivalis* in the oropharyngeal cavity.^15^ In response to such pathogen/damage-associated molecular pattern-stimulating events (arising not only from infections but also from ischemia and trauma, with ischemia also being caused by COVID-19), amyloid-β (Aβ) is released as an early responder peptide propagating an ensuing innate immunity cascade, manifesting as production of pro-inflammatory cytokine and neuroinflammation-associated peptides, including interleukins (IL1R1, IL3, IL4, IL6, IL10, IL12), interferons (IFNγ), macrophage inflammatory proteins (MIP1α, MIP1β), and TNF-α. This sustained released of pro-inflammatory cytokines and activated microglia heralds a dyshomeostatic imbalance between pro- and anti-inflammatory processes in brain creating a substrate for AD’s chronic progressive neuronal death over subsequent years.

## Could COVID-19 Be a Risk Factor for Alzheimer’s?

An analysis of the pathogenesis and disease progression of COVID-19 leads to the possibility that Alzheimer’s disease may emerge as a long-term (N^th^ wave) consequence of COVID-19. The quality of biological and clinical evidence upon which this analysis is based is understandably variable given the urgency and recency of COVID-19. Available data range from well-controlled *in vitro* and *in vivo* experiments to clinical research in which case studies and observational studies dominate and randomized studies are still comparatively few. Nonetheless, consistencies and emerging trends do appear, permitting a number of postulates and conclusions to be proposed.

Since COVID-19 and AD are both neuroimmunological diseases, neuroinflammation plays an early and central etiopathogenic role in both diseases. The viral RNA of SARS-CoV-2 constitutes a PAMP capable of provoking a pro-inflammatory immune reaction in brain. Moreover as discussed above, SARS-CoV-2 is a neurotropic virus present in both CSF and brain tissue, while COVID-19 is a neuroinflammatory disease sometimes associated with a pro-inflammatory cytokine storm in brain and the development of new symptoms after resolution of the acute phase (Long-COVID). In accordance with recent conceptual advances in Alzheimer’s research, the immune response and excessive inflammation of COVID-19 could readily predispose to neurodegeneration or Alzheimer’s disease. Moreover, this predisposition is possible across the full age spectrum of patients afflicted with COVID-19: Elderly individuals are more susceptible to both severe SARS-CoV-2 infection and neurodegenerative disorders; younger patients have a 20-40 year runway ahead of them affording ample time for a full expression of neuroinflammation as neurodegeneration. Because brain inflammation may initiate and accompany a multiplicity of neurodegenerative disorders and may contribute to major brain diseases, the neurological and psychiatric sequelae of COVID-19 need to be rigorously monitored; the possibility that an N^th^ wave of COVID-19-could manifest as AD demands public health awareness and scrutiny.

## Conclusions

Regrettably, we are still in the early days of COVID as a globally present pandemic and public health crisis. The full story of COVID-19 has yet to be written, and there shall probably be future new variants and future new waves. As argued herein, a reasonable case can be put forth postulating that Alzheimer’s disease could be a long-term consequence of COVID-19-triggered neuroinflammation. Though this is speculative, its potential public health implications are sufficiently immense to warrant more detailed monitoring and study.

As of August 2021, 215 million COVID-19 cases had been confirmed worldwide, with more than 4.5 million deaths, establishing it as one of the deadliest pandemics in human history. With a global prevalence of 50 million people living with dementia (and a new case being diagnosed every four seconds globally), AD/dementia is also a pandemic in its own right, often listed as one of the “other pandemics” co-existing with COVID-19. COVID-19 is infectious with high prevalence and an acute case fatality rate ranging from 0.1–25% depending upon patient age and living environment; AD has lower prevalence, but is uniformly incurable and ultimately fatal, contributing in some capacity to the death of the majority of its victims. Though people with AD are particularly susceptible to COVID-19 as evidenced by international experiences in long-term care facilities, dementia and COVID-19 have otherwise been regarded as essentially parallel pandemics. Given the already large (and still escalating) prevalence of COVID-19, even a 10–20% chronic SARS-CoV-2-mediated neuroinflammation rate might conceivably lead to a doubling of the global burden of dementia within decades – currently, the annual global cost of dementia is $1 trillion dollars, an amount expected to double to $2 trillion by 2030 even in the absence of any possible COVID-19-related caseload surge. An N^th^ wave of COVID-19 presenting as AD or other types of dementia would represent a devastating cross-over point between these two previously parallel paths – a merger with immense public health and socioeconomic consequences.
